# N6-methyladenosine (m6A) RNA methylation regulator *SNRPC* is a prognostic biomarker and is correlated with immunotherapy in hepatocellular carcinoma

**DOI:** 10.1186/s12957-021-02354-8

**Published:** 2021-08-13

**Authors:** Jihao Cai, Minglei Zhou, Jianxin Xu

**Affiliations:** 1 The Second Clinical Medical College of Nanchang University, Nanchang, China; 2 School of Computer Science and Technology of Shandong University of Technology, Zibo, China

**Keywords:** *SNRPC*, Hepatocellular carcinoma, Prognosis, Immune checkpoint, Immunotherapy

## Abstract

**Background:**

Hepatocellular carcinoma (HCC) is one of the most common malignancies in the world, and due to its complex pathogenic factors, its prognosis is poor. N6-methyladenosine (m^6^A) RNA methylation plays an important role in the tumorigenesis, progression, and prognosis of many tumors. The m^6^A RNA methylation regulator small nuclear ribonucleoprotein polypeptide C (*SNRPC*), which encodes one of the specific protein components of the U1 small nuclear ribonucleoprotein (snRNP) particle, has been proven to be related to the prognosis of patients with HCC. However, the effect of *SNRPC* on the tumor microenvironment and immunotherapy in HCC remains unclear.

**Case presentation:**

The HCC RNA-seq profiles in The Cancer Genome Atlas (TCGA) and International Cancer Genome Consortium (ICGC) databases, including 421 LIHC and 440 LIRI-JP samples, respectively, were used in this study. Both the expression of *SNRPC* in HCC was upregulated in the TCGA and ICGC databases compared to normal tissues. Next, the expression of *SNRPC* was validated as a risk factor for prognosis by Kaplan-Meier analysis and employed to establish a nomogram with T pathologic stage. By gene set variation (GSVA) analysis and gene set enrichment (GSEA) analysis, we found that *SNRPC* was mainly related to protein metabolism and the immune process. Furthermore, the estimation of stromal and immune cells in malignant tumor tissues using expression (ESTIMATE), microenvironment cell population counter (MCP-counter), and single sample GSEA (ssGSEA) algorithms revealed that the high-*SNRPC* group had a lower stromal score, lower abundance of endothelial cells and fibroblasts, and lower immune infiltration. Ultimately, a tumor immune dysfunction and exclusion (TIDE) analysis revealed that patients in the low-*SNRPC* group may be more sensitive to immune checkpoint inhibitor therapy.

**Conclusion:**

*SNRPC* could serve as a promising prognostic and immunotherapeutic marker in HCC and might contribute to new directions and strategies for HCC treatment.

## Background

Liver cancer was the third leading cause of cancer death worldwide in 2020 [1]. Hepatocellular carcinoma (HCC) is the predominant form of primary liver cancer and accounts for 75–85% of cases. Chronic infection with hepatitis B virus (HBV) or hepatitis C virus (HCV), heavy alcohol intake, type 2 diabetes, and smoking have been related to the occurrence of HCC [1]. More than 1 million liver cancer deaths are estimated by 2030 [2]. Although surgical resection of early HCC is still the preferred treatment, the recurrence rate at 5 years can be as high as 70% [3]. In addition, even if liver transplantation can reduce the recurrence rate, the number of available donor organs is always insufficient relative to the demand [4]. Moreover, the majority (> 70%) of patients with advanced disease do not benefit from surgery [5]. For these patients, immunotherapy is a potential option. In recent years, emerging immunotherapies, such as immune checkpoint blockade and chimeric antigen receptor t cell (CAR-T) immunotherapy, have shown better therapeutic outcomes for some tumors [6, 7]. Nevertheless, responses to immunotherapy typically occur in a minority of patients, ranging from 20 to 50% depending on the tumor type [8]. Thus, it is especially important to identify candidate biomarkers to target patients who have the greatest likelihood of benefiting from immunotherapy [8]. In 2017, the Food and Drug Administration (FDA) approved the PD-1 inhibitor nivolumab (OPDIVO®), which marked the beginning of the immunotherapy era for HCC [9]. Compared with certain tumors, immunotherapy for liver cancer is still in its infancy. Additionally, knowledge of the biomarkers for predicting the responses or primary resistance to immunotherapeutic agents is crucial for establishing immunotherapy criteria for HCC patients in the future.

Genome-wide analysis of mRNA expression profiles has been used to screen prognostic and treatment-related markers in many cancers in recent years [10–13]. The N6-methyladenosine (m6A) methylation modification occurs at the 6th N atom of adenine, and it is the most common posttranscriptional modification on RNA and mediates > 60% of RNA methylation [14, 15]. Studies have shown that m6A RNA methylation plays an important role in tumor occurrence, development, and prognosis [16]. As an important m^6^A RNA methylation regulator, small nuclear ribonucleoprotein polypeptide C (*SNRPC*) encodes one of the specific protein components of the U1 small nuclear ribonucleoprotein (snRNP) particle, which is required for the formation of the spliceosome [17]. A recent study showed that *SNRPC* has the potential to promote HCC cell motility by inducing epithelial-mesenchymal transition [18]. In addition, *SNRPC* also contributes to sex bias in systemic autoimmune diseases and plays a role in spinal muscular atrophy pathogenesis [19, 20]. However, to our knowledge, the interaction between *SNRPC* and immunotherapy in HCC has not been reported. Therefore, in this study, we aimed to analyze the potential of *SNRPC* as a prognostic biomarker and predictor of immunotherapy responses in HCC to provide reference data for the clinical treatment of HCC.

In this study, we found that *SNRPC* expression was upregulated in HCC samples compared to normal samples. Next, the expression of *SNRPC* was validated as a risk factor for prognosis by a Kaplan-Meier analysis and employed to establish a nomogram with T pathologic stage. Furthermore, we investigated the correlation between *SNRPC* and immunotherapeutic reactions through a tumor immune dysfunction and exclusion (TIDE) analysis. These findings may contribute to new directions and strategies for HCC treatment.

## Materials and methods

### Public transcriptome profiling

In this study, we used 861 HCC cases from two public databases. After excluding samples without clinical annotation and survival information, 421 LIHC samples (50 normal samples labeled with 11A and 364 HCC samples labeled with 01A) were obtained from The Cancer Genome Atlas (TCGA) database and used as a training set. Additionally, 440 LIRI-JP cases (197 normal and 243 HCC) were obtained from the International Cancer Genome Consortium (ICGC) database and used as a validation set. The detailed clinical data of these samples are listed in Table 1. Moreover, both the expression data from the TCGA and ICGC databases were standardized by the log2 function in R.

**Table 1:** Sex, tumor stage, and age of the samples from the ICGC database

	Number	Ratio
**Gender**		
Female	68	0.261538462
Male	192	0.738461538
**Stage**		
Stage1	40	0.153846154
Stage2	117	0.45
Stage3	80	0.307692308
Stage4	23	0.088461538
**Age**		
> 60	205	0.788461538
< 60	55	0.211538462

### Validation of the expression of *SNRPC* in HCC based on the TCGA and ICGC databases

To further investigate the expression of *SNRPC* in HCC, the Wilcoxon test was used to verify the significant difference in mRNA expression levels of *SNRPC* between the tumor and normal tissue samples based on the TCGA and ICGC databases. Moreover, the expression of *SNRPC* was further examined between 50 HCC tissue samples and 50 paired paracancerous tissue samples from the TCGA database (the ICGC database does not include paired cancer tissue and paracancerous tissue). Finally, the protein expression of *SNRPC* between HCC tissues and normal tissues was verified in the Human Protein Atlas (HPA) database (https://www.proteinatlas.org/).

### Survival analysis of *SNRPC*

To evaluate whether *SNRPC* could impact the survival probability of HCC, all of the HCC samples in the TCGA and ICGC databases were divided into a high-*SNRPC* group and a low-*SNRPC* group based on the median expression value of *SNRPC*. Subsequently, survival probabilities were analyzed by a Kaplan-Meier analysis and log-rank test [21], with a *p* value < 0.05 considered statistically significant. In addition, time-dependent receiver operating characteristic (ROC) curves were plotted to assess the prognostic prediction performance of *SNRPC*, and the areas under the curve (AUCs) for overall survival (OS) were calculated using the survival ROC R package [22].

### Construction of a nomogram for prognostic prediction

For better clinical application of *SNRPC*, we established a nomogram by univariate and multivariate Cox regression analysis to evaluate the probability of 1-, 3-, and 5-year OS for HCC patients via the rms R package [23] by integrating the clinical factors and *SNRPC* expression.

### Functional annotation

To investigate the potential biological function of *SNRPC* in HCC, all of the differentially expressed genes between the high-*SNRPC* group and the low-*SNRPC* group in the TCGA database were ranked based on log_2_FC values. Then, a gene set enrichment analysis (GSEA) was implemented using the clusterProfiler package [24] to estimate the pathway activity changes in the above two groups. Similarly, the gene set variation (GSVA) package [25] was utilized to find the predominant pathways associated with *SNRPC*. Terms with values of FDR < 0.25 and *p* < 0.05 were retained.

### ESTIMATE analysis

The Estimation of STromal and Immune cells in MAlignant Tumor tissues using Expression (ESTIMATE) algorithm can calculate immune scores and stromal scores via the estimate package in R [26], allowing us to quantify the immune and stromal components in a tumor. Therefore, the differences in immune scores, stromal scores, and tumor purity between the high-*SNRPC* group and the low-*SNRPC* group in the TCGA database were assessed by the Wilcoxon test.

### Analysis of the tumor microenvironment

To gain further insights into the composition of the tumor microenvironment between the high-*SNRPC* group and low-*SNRPC* group in the TCGA database, a microenvironment cell population counter (MCP-counter) was used to quantify the numbers of immune cells, fibroblasts, and epithelial cells from each HCC sample based on marker genes [27]. Then, the single sample GSEA (ssGSEA) algorithm was used to calculate the immune infiltration of each sample, and significant differences in immune cell numbers were identified by analysis of variance (ANOVA).

### Tumor immune dysfunction and exclusion analysis

Immune checkpoint pathways are associated with tumor immune escape. Therefore, immune checkpoint inhibitors can enhance anticancer immunity. Thus, we also employed the tumor immune dysfunction and exclusion (TIDE) algorithm [28] and subclass mapping [29] to predict the clinical response to immune checkpoint inhibitors between the high-*SNRPC* group and the low-*SNRPC* group. Moreover, we also calculated the correlation between *SNRPC* expression and TIDE scores.

### Statistical analysis

All statistical tests were performed using R (version 3.5.2) software. Continuous data were analyzed using the Wilcoxon test or ANOVA. A *p* value of less than 0.05 was considered statistically significant in all analyses.

## Results

### *SNRPC* is upregulated in HCC

**Fig. 1:**
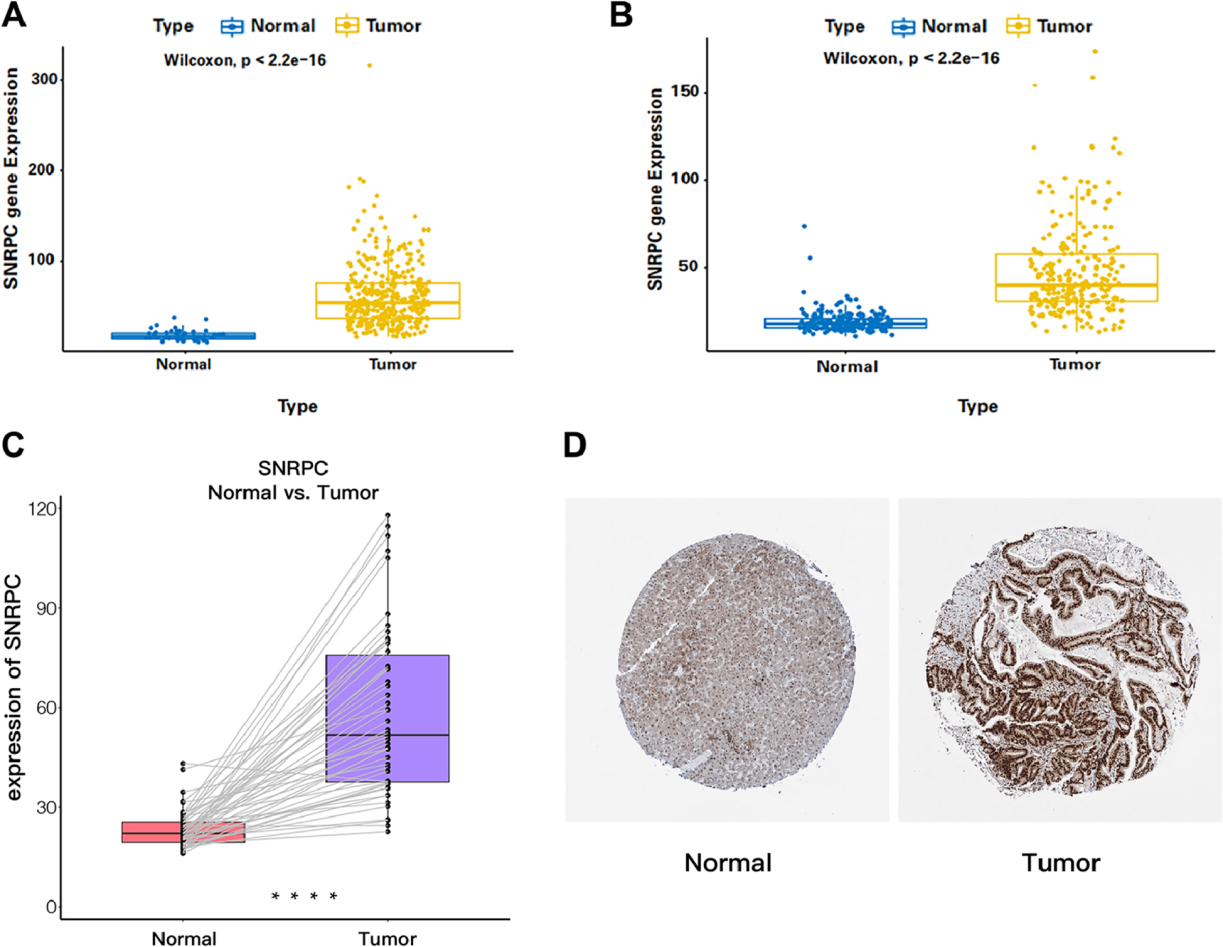
**A** Expression of *SNRPC* in the TCGA-LIHC dataset. **B** Expression of *SNRPC* in the ICGC-LIRI dataset. **C** Expression of *SNRPC* between paired HCC and paracancerous tissues in the TCGA database. **D** Protein expression of *SNRPC* between HCC tissues and normal tissues in the HPA database

To further investigate the expression of *SNRPC* in HCC, *SNRPC* expression between the HCC and normal samples was analyzed using data from the TCGA and ICGC databases. Interestingly, the results of both the TCGA and ICGC databases suggested that *SNRPC* expression was upregulated in patients with HCC compared to normal samples (Fig. 1A, B). Moreover, the analysis of paired HCC and paracancerous tissues also revealed that *SNRPC* expression was upregulated in HCC tissues (Fig. 1C). Finally, a deeper staining level of *SNRPC* in HCC tissues compared to normal tissues revealed a higher protein expression of *SNRPC* in HCC (Fig. 1D).

### *SNRPC* is related to the survival of HCC

**Fig. 2:**
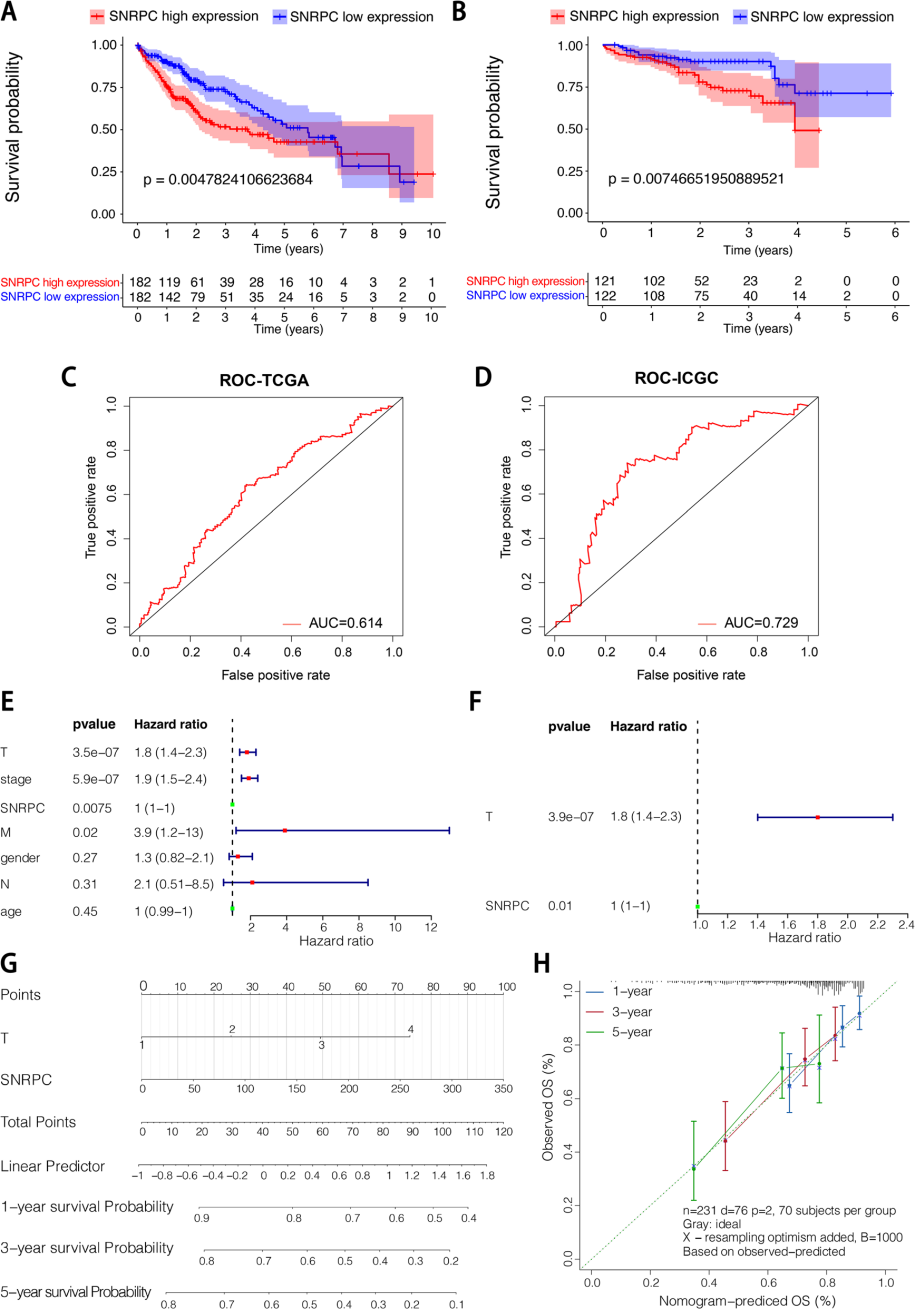
**A** KM survival curve of total survival time based on the *SNRPC* in the TCGA-LIHC dataset. **B** KM survival curve of total survival time based on the *SNRPC* in the ICGC-LIRI dataset. **C** ROC curve using samples from the TCGA database. **D** ROC curve using samples from the ICGC database. **E** Univariate Cox regression analysis. **F** Multivariate Cox regression analysis. **G** Nomogram for HCC 1-, 3- and 5-year prognosis. **H** Calibration curve

A Kaplan-Meier survival curve was drawn, and the results showed that in both the TCGA and ICGC databases, the low-*SNRPC* groups had a high survival probability relative to the high-*SNRPC* groups (*p* < 0.05) (Fig. 2A, B). In addition, as shown in the ROC curves, the AUCs of *SNRPC* were 0.614 and 0.729 in the TCGA and ICGC databases, respectively (Fig. 2C, D).

### Construction of a nomogram for predicting HCC prognosis

The univariate and multivariate Cox regression analysis results suggested that the pathological T stage and *SNRPC* were significantly correlated with the prognosis of HCC (Fig. 2E, F). Therefore, a nomogram including T clinical factors and *SNRPC* expression values was constructed for predicting the 1-, 3-, and 5-year survival probabilities of HCC using samples from the TCGA database (Fig. 2G). A calibration curve demonstrated that our nomogram could accurately estimate the mortality of HCC (Fig. 2H).

### Identification of *SNRPC*-related functional annotation

**Fig. 3:**
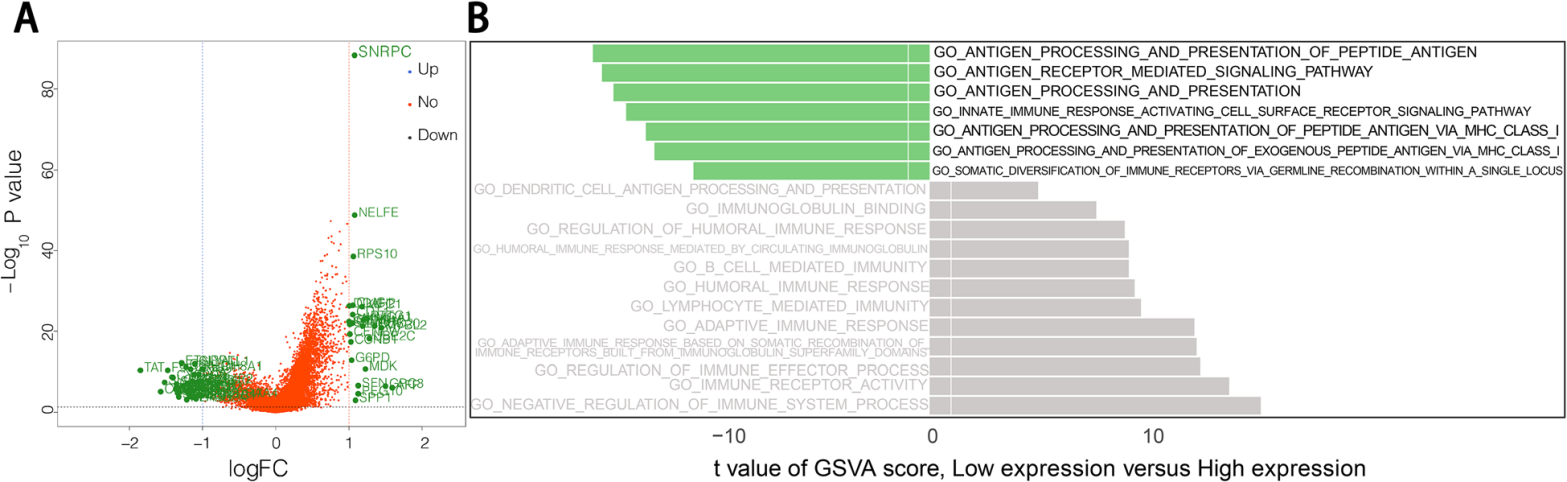
**A** Volcano map showing the low- and high-*SNRPC* groups. **B** Nineteen immune-related items enriched in the high- and low-*SNRPC* groups by GSVA

To gain insights into the potential functions related to *SNRPC*, we first identified 64 differentially expressed genes (DEGs) between the HCC samples of the high-*SNRPC* group and the low-*SNRPC* group (Fig. 3A). We found that catalytic activity-related biological processes, including catalytic_activity_acting_on_DNA, catalytic_step_2_spliceosome, and catalytic_activity_acting_on_RNA, were mainly activated in the high-*SNRPC* group (Table 2). Conversely, metabolism-related biological processes, including alcohol metabolism, amino acid metabolism, lipid metabolism, etc., were mainly activated in the low-*SNRPC* group (Table 2). Among the Kyoto Encyclopedia of Genes and Genomes (KEGG) pathways, the cell cycle-, spliceosome-, ribosome-, and complement-related pathways were mainly activated in the high-*SNRPC* group while the metabolism-related pathways were mainly activated in the high-*SNRPC* group (Table 3). In addition, the GSVA results suggested that a large number of immune-related processes were disordered. For example, antigen processing-related biological processes were mainly upregulated in the high-*SNRPC* group while immune response-related biological processes were mainly upregulated in the low-*SNRPC* group (Fig. 3B, Table 4).

**Table 2:** Top 10 GO terms annotated in the high- and low-*SNRPC* expression groups

Terms	NES	*p* value	*p*-adjust
GO_ACUTE_INFLAMMATORY_RESPONSE	− 2.320055852	1.00E-10	5.02E-09
GO_ALCOHOL_METABOLIC_PROCESS	− 1.785043835	1.00E-10	5.02E-09
GO_ALPHA_AMINO_ACID_CATABOLIC_PROCESS	− 2.682453887	1.00E-10	5.02E-09
GO_ALPHA_AMINO_ACID_METABOLIC_PROCESS	− 2.355743228	1.00E-10	5.02E-09
GO_BLOOD_MICROPARTICLE	− 2.56496589	1.00E-10	5.02E-09
GO_CATALYTIC_ACTIVITY_ACTING_ON_DNA	1.82665719	1.00E-10	5.02E-09
GO_CATALYTIC_ACTIVITY_ACTING_ON_RNA	1.681035365	1.00E-10	5.02E-09
GO_CATALYTIC_STEP_2_SPLICEOSOME	1.970594215	1.00E-10	5.02E-09
GO_CELLULAR_AMINO_ACID_CATABOLIC_PROCESS	− 2.691409978	1.00E-10	5.02E-09
GO_CELLULAR_LIPID_CATABOLIC_PROCESS	− 2.125968674	1.00E-10	5.02E-09

**Table 3:** Top 10 KEGG terms enriched in the high- and low-*SNRPC* expression groups

Terms	NES	*p* value	*p*-adjust
KEGG_CELL_CYCLE	1.858825048	6.01E-10	1.38E-08
KEGG_COMPLEMENT_AND_COAGULATION_CASCADES	− 2.985586938	1.00E-10	3.07E-09
KEGG_DNA_REPLICATION	1.97287282	2.06E-08	3.78E-07
KEGG_DRUG_METABOLISM_CYTOCHROME_P450	− 2.545914368	1.00E-10	3.07E-09
KEGG_FATTY_ACID_METABOLISM	− 2.639833598	1.00E-10	3.07E-09
KEGG_PPAR_SIGNALING_PATHWAY	− 2.409646016	5.09E-10	1.34E-08
KEGG_RETINOL_METABOLISM	− 2.808138608	1.00E-10	3.07E-09
KEGG_RIBOSOME	2.545828247	1.00E-10	3.07E-09
KEGG_SPLICEOSOME	2.04347142	1.00E-10	3.07E-09
KEGG_VALINE_LEUCINE_AND_ISOLEUCINE_DEGRADATION	− 2.486033153	4.87E-09	9.96E-08

**Table 4:** Nineteen immune-related items enriched in the high- and low-*SNRPC* expression groups by GSVA

Type	Average GSVA score		*p*
	Low_*SNRPC*	High_*SNRPC*	
GO_HUMORAL_IMMUNE_RESPONSE	0.243174558	− 0.171333299	< 2.2e−16
GO_REGULATION_OF_HUMORAL_IMMUNE_RESPONSE	0.217516904	− 0.152275441	< 2.2e−16
GO_HUMORAL_IMMUNE_RESPONSE_MEDIATED_BY_CIRCULATING_IMMUNOGLOBULIN	0.240056081	− 0.194481039	< 2.2e−16
GO_REGULATION_OF_IMMUNE_EFFECTOR_PROCESS	0.231855592	− 0.168855904	< 2.2e−16
GO_ADAPTIVE_IMMUNE_RESPONSE_BASED_ON_SOMATIC_RECOMBINATION_OF_IMMUNE_RECEPTORS_BUILT_FROM_IMMUNOGLOBULIN_SUPERFAMILY_DOMAINS	0.225524044	− 0.17393338	< 2.2e−16
GO_B_CELL_MEDIATED_IMMUNITY	0.240056081	− 0.194481039	< 2.2e−16
GO_INNATE_IMMUNE_RESPONSE_ACTIVATING_CELL_SURFACE_RECEPTOR_SIGNALING_PATHWAY	− 0.261145258	0.190182919	< 2.2e−16
GO_ADAPTIVE_IMMUNE_RESPONSE	0.227846975	− 0.175474417	< 2.2e−16
GO_LYMPHOCYTE_MEDIATED_IMMUNITY	0.234004969	− 0.178173068	< 2.2e−16
GO_NEGATIVE_REGULATION_OF_IMMUNE_SYSTEM_PROCESS	0.229845123	− 0.166309991	< 2.2e−16
GO_IMMUNE_RECEPTOR_ACTIVITY	0.196420292	− 0.141311054	< 2.2e−16
GO_SOMATIC_DIVERSIFICATION_OF_IMMUNE_RECEPTORS_VIA_GERMLINE_RECOMBINATION_WITHIN_A_SINGLE_LOCUS	− 0.222668922	0.155772825	1e−13
GO_IMMUNOGLOBULIN_BINDING	0.186800511	− 0.125978607	< 2.2e−16
GO_ANTIGEN_PROCESSING_AND_PRESENTATION_OF_EXOGENOUS_PEPTIDE_ANTIGEN_VIA_MHC_CLASS_I	− 0.237508396	0.180189519	< 2.2e−16
GO_ANTIGEN_PROCESSING_AND_PRESENTATION_OF_PEPTIDE_ANTIGEN	− 0.259010315	0.210540976	< 2.2e−16
GO_ANTIGEN_PROCESSING_AND_PRESENTATION_OF_PEPTIDE_ANTIGEN_VIA_MHC_CLASS_I	− 0.240473386	0.183322272	< 2.2e−16
GO_ANTIGEN_PROCESSING_AND_PRESENTATION	− 0.230240714	0.187319947	< 2.2e−16
GO_ANTIGEN_RECEPTOR_MEDIATED_SIGNALING_PATHWAY	− 0.231518181	0.178373361	< 2.2e−16
GO_DENDRITIC_CELL_ANTIGEN_PROCESSING_AND_PRESENTATION	0.180625588	0.005442914	6.8e−07

### ESTIMATE analysis

**Fig. 4:**
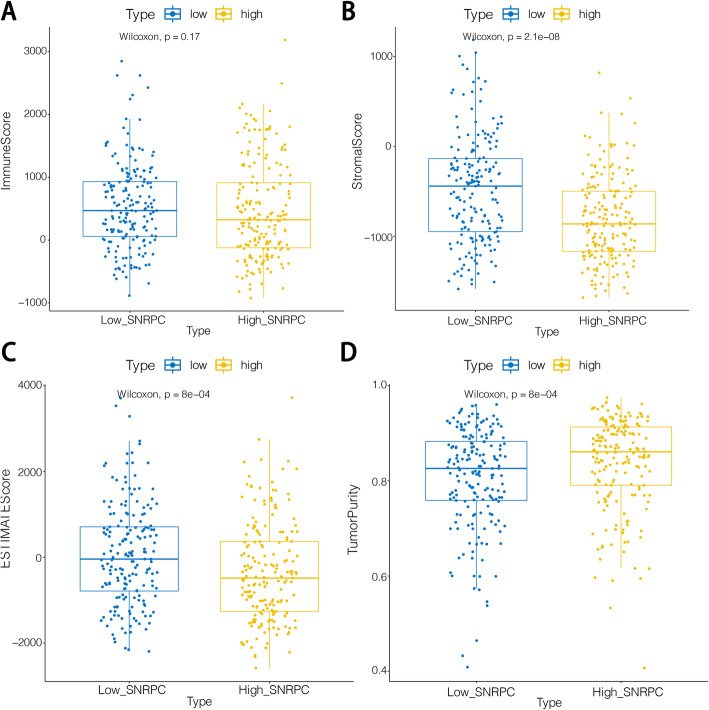
**A** Box diagram showing the immune scores in the high- and low-*SNRPC* groups. **B** Box diagram showing the stromal scores in the high- and low-*SNRPC* groups. **C** Box diagram demonstrating the ESTIMATE scores in the high- and low-*SNRPC* groups. **D** Box diagram demonstrating the tumor purity in the high- and low-*SNRPC* groups

In our study, we found that except for the immune scores, the stromal scores, ESTIMATE scores, and tumor purity were significantly different between the high- and low-*SNRPC* groups (Fig. 4). We also found that a lower stromal score and higher tumor purity were associated with poor survival in HCC patients.

### Correlation between *SNRPC* expression and tumor microenvironment

**Fig. 5:**
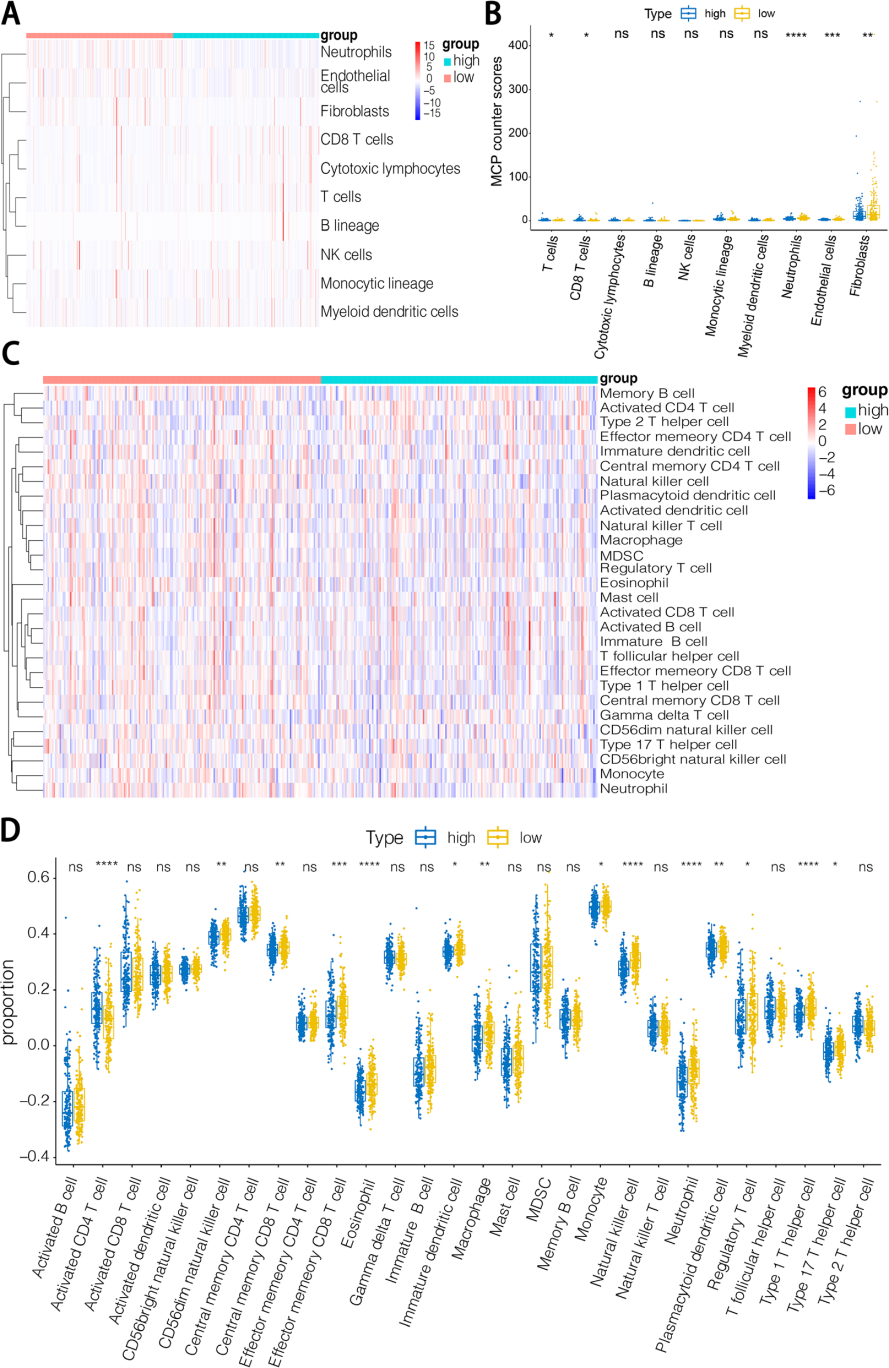
**A** Abundance of TME cells between the high- and low-*SNRPC* groups calculated by MCP-counter. **B** Box plot showing the proportion of TME cells between the high- and low-*SNRPC* groups analyzed by MCP-counter. **C**, **D** Heat map and box plot showing the infiltration of immune cells between the high- and low-*SNRPC* groups calculated by ssGSEA

MCP-counter and ssGSEA analyses were conducted to clarify the abundance of various cells in the tumor microenvironment (TME). Immune cells, such as T cells, CD8^+^T cells, neutrophils, and stromal cells, such as endothelial cells and fibroblasts, were significantly different between the high- and low-*SNRPC* groups. Among these cell types, neutrophils, endothelial cells, and fibroblasts were increased in the low-*SNRPC* group while other types were decreased (Fig. 5A, B). Through the ssGSEA, additional subtypes of immune cells were counted. In the low-*SNRPC* group, only activated CD4^+^T cells were significantly decreased while CD56dim natural killer cells, central memory CD8^+^T cells, effector memory CD8^+^T cells, eosinophils, immature dendritic cells, macrophages, memory B cells, monocytes, natural killer T cells, natural killer T cells, plasmacytoid dendritic cells, regulatory T cells, type 1 T helper cells, and type 17 T helper cells were all increased (Fig. 5C, D).

### *SNRPC* expression could predict the immunotherapeutic response of HCC

**Fig. 6:**
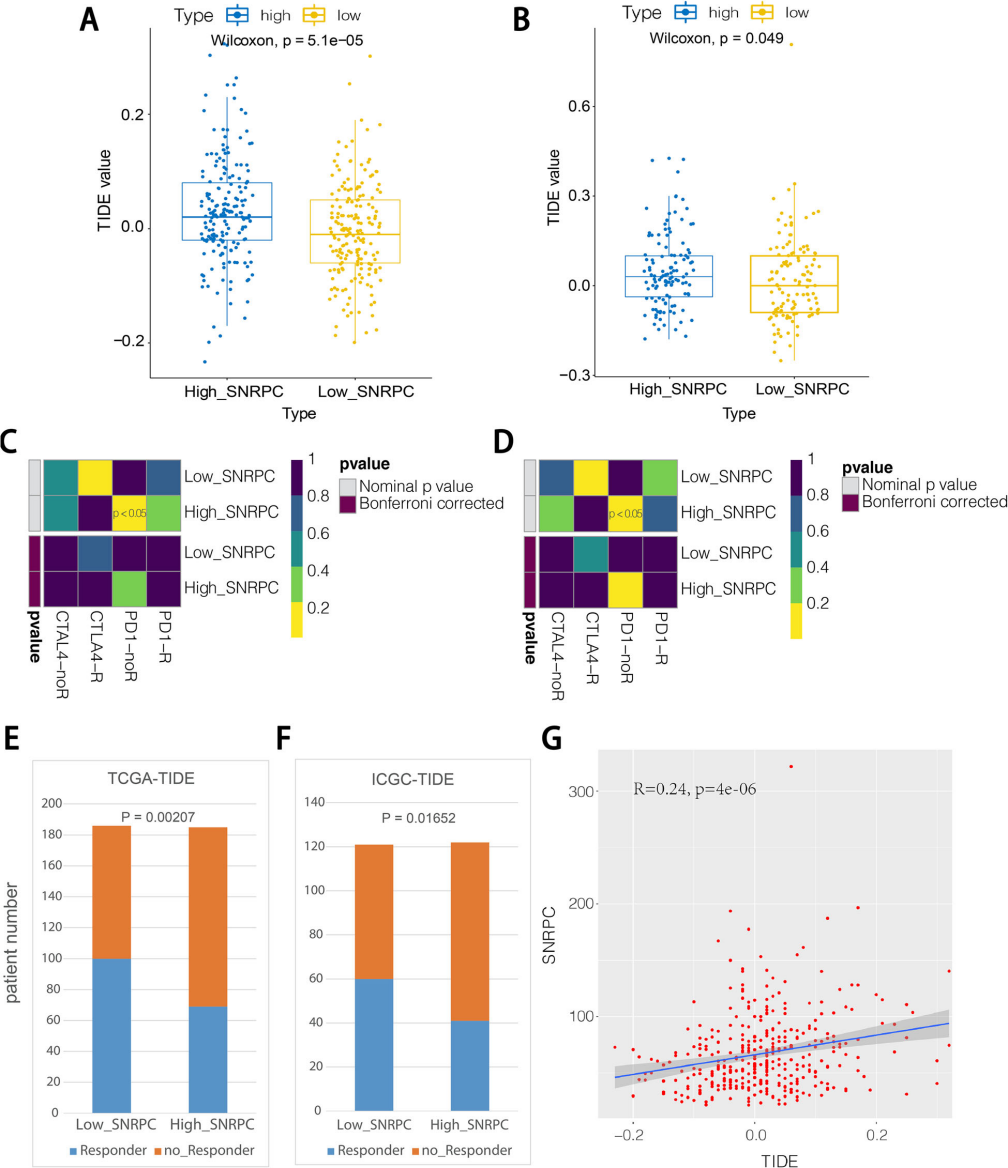
**A** TIDE scores of the high- and low-*SNRPC* groups using samples from the TCGA database. **B** TIDE scores of the high- and low-*SNRPC* groups using data from the ICGC database. **C** Immunotherapy responses between the high- and low-*SNRPC* groups in the TCGA database. **D** Immunotherapy responses between the high- and low-*SNRPC* groups in the ICGC database. **E** Proportion of patients who responded to immunotherapy in the high-*SNRPC* and low-*SNRPC* groups in the TCGA. **F** Proportion of patients who respond to immunotherapy in the high-*SNRPC* and low-*SNRPC* groups in the ICGC. **G** Correlation analysis between *SNRPC* expression and TIDE score

The TIDE score is used to predict the efficacy of anti-PD1 and anti-CTLA4 treatments [27]. As shown in Fig. 6A, B, both the high-*SNRPC* groups showed higher TIDE scores in the TCGA and ICGC databases. Consistently, both the high-*SNRPC* groups showed no response to anti-PD1 therapy in the TCGA and ICGC databases by subclass mapping analysis (Fig. 6C, D). Furthermore, both the low-*SNRPC* groups presented more patients with the response to immune checkpoint inhibitor therapy in the TCGA and ICGC databases (Fig. 6E, F). More importantly, the correlation analysis suggested that the expression of *SNRPC* was positively correlated with the TIDE scores (Fig. 6G)*.* Thus, these findings revealed that patients in the low-*SNRPC* group may be more sensitive to immune checkpoint inhibitor therapy.

## Discussion

HCC accounts for 75–85% of liver cancers and is also the third leading cause of cancer-related death globally, which has a poor prognosis [1]. Studies have shown that multiple genes play an important role in HCC progression [30–34]. Currently, immunotherapy has already become a viable option for advanced patients. However, not all patients show complete responses to such immunotherapies [35]. As a result, a reliable biomarker for predicting HCC prognosis and immunotherapy response is urgently needed.

In the present study, we further analyzed the role of *SNRPC* in HCC. *SNRPC* encodes one of the specific protein components of U1snRNP, which plays an essential role in the formation of the spliceosome. U1snRNP is reported to suppress the migration and invasion of multiple types of tumor cells [36]. Additionally, genes of the snRNP polypeptide family also exert an important role in the tumorigenesis and prognosis of cancers [37, 38]. For example, the high expression of snRNP polypeptide G, which is another indispensable component in the formation of snRNPs, is positively associated with the occurrence, progression and severity of breast, lung, and colon cancers [39–41]. Moreover, the snRNP polypeptide N is highly expressed in colorectal cancer tissues and involved in the progression of colorectal cancer [42]. A recent study demonstrated that *SNRPC* has the potential to enhance HCC cell motility by promoting epithelial-mesenchymal transition, which might contribute to a poor prognosis [18]. However, few studies have focused on the role of *SNRPC* in the immunotherapy of tumors. Therefore, this study aimed to reveal the effect of *SNRPC* on the prognosis and immunotherapy of HCC. In our study, we first found that *SNRPC* was overexpressed in HCC and that higher expression of *SNRPC* was associated with a poorer prognosis. Then, we constructed a nomogram containing T clinical factors and *SNRPC* expression values, which can precisely predict 1-, 3-, and 5-year survival probabilities. To gain detailed insights into the mechanisms of *SNRPC*, we performed functional analyses and found that *SNRPC* was mainly related to protein metabolism and the immune process. Interestingly, a TME analysis indicated that natural killer cells, CD8^+^T cells, macrophages, neutrophils, endothelial cells, and fibroblasts were less infiltrated in tissues with high *SNRPC* expression. These results implied that both stromal cells and immune cells were abundant, which was beneficial for HCC prognosis. Ultimately, we employed the TIDE analysis method to predict the efficacy of anti-PD1 and anti-CTLA4 treatments. The results showed that the high-*SNRPC* group failed to respond to immune checkpoint inhibitor (ICI) therapy, especially to a PD-1 inhibitor.

At present, many clinical prognostic biomarkers are available for HCC, such as alpha-fetoprotein (AFP), alpha-fetoprotein lens culinaris agglutin-3 (AFP-L3), and des-γ-carboxy prothrombin (DCP). The relationship between AFP and prognosis has been extensively studied [43]. Increasing AFP values are associated with lower survival and a higher tumor recurrence rate in patients at very early or early stages as well as poor prognosis in patients undergoing advanced HCC [43]. By comparison, overexpressed *SNRPC* is correlated with low infiltration of immune cells and stromal cells, poor prognosis, and poor immunotherapy response. However, *SNRPC* expression remains relatively complex to detect compared with AFP, which can be detected in serum. We hope that in the future, *SNRPC* could be used in combination with other biomarkers for the clinical staging system and prognosis prediction of HCC.

At present, numerous biomarkers have been employed to select candidates for immunotherapy. PD-L1 expression is the most widely adopted predictor of the immunotherapeutic response rate. High PD-L1 expression is associated with an increased response rate and clinical benefit in anti-PD-1/anti-PD-L1 therapy [44]. However, PD-L1 expression is not associated with treatment response in some patients. Additionally, the TIDE score can also directly guide the use of ICIs. Patients with higher TIDE scores have higher antitumor immune escape opportunities, resulting in lower ICI treatment response rates [28]. Moreover, the TIDE score has been shown to have higher accuracy than the PD-L1 expression level in predicting the survival outcome of cancer patients treated with ICI agents [45–47]. In the current study, the TIDE analysis revealed that patients in the low-*SNRPC* group may be more sensitive to immune checkpoint inhibitor therapy. Therefore, we speculated that *SNRPC* might be associated with the immunotherapeutic response of HCC.

Recently, novel strategies have been identified to enhance the effect of anti-PD-1/PD-L1 by promoting T cell infiltration, such as an anti-TGF-β/PD-L1 bispecific antibody and anti-PD-1 combining anti-CTLA-4 [48, 49]. Combining immunotherapy with anti-VEGF agents also displayed a good therapeutic effect in some HCC cases. Consequently, *SNRPC* might contribute to improving the effectiveness of anti-PD-1/PD-L1 by regulating the expression of *SNRPC*.

## Conclusion

In this study, we showed that the HCC biomarker *SNRPC* was overexpressed in tumor tissues and linked to a worse prognosis in patients with HCC. Functional analysis showed that *SNRPC* was mainly related to protein metabolism and the immune process. In addition, we found that upregulated *SNRPC* may be associated with a less sensitive response to immune checkpoint inhibitor therapy. Therefore, *SNRPC* may be a promising prognostic and immunotherapeutic marker for HCC. Nevertheless, we only included anti-PD1 and anti-CTLA4 treatments in our TIDE analysis rather than considering more promising immunotherapy methods such as combination therapies. In addition, we did not choose a proposed cutoff for predicting poor prognosis and immunotherapeutic response. Hence, follow-up experiments and clinical validation are extremely necessary.

## Data Availability

The datasets analyzed during the current study are available in the TGCA [https://portal.gdc.cancer.gov], ICGC [https://dcc.icgc.org/] public database, and HPA [https://www.proteinatlas.org/].

## Abbreviations

HCC: Hepatocellular carcinoma
HBV: Hepatitis B virus
HCV: Hepatitis C virus
CAR-T: Chimeric antigen receptor T cell
FDA: Food and Drug Administration
m6A: N6-methyladenosine
*SNRPC*: Small nuclear ribonucleoprotein polypeptide C
snRNP: Small nuclear ribonucleoprotein
TIDE: Tumor immune dysfunction and exclusion
TCGA: The Cancer Genome Atlas
ICGC: International Cancer Genome Consortium
HPA: Human Protein Atlas
ROC: Receiver operating characteristic
AUCs: Areas under the curve
OS: Overall survival
GSEA: Gene set enrichment analysis
GSVA: Gene set variation
ESTIMATE: Estimation of STromal and Immune cells in MAlignant Tumor tissues using Expression
MCP-counter: Microenvironment Cell Population counter
ssGSEA: Single sample GSEA
ANOVA: Analysis of variance
DEGs: Differential expression genes
GO: Gene ontology
KEGG: Kyoto Encyclopedia of Genes and Genomes
TME: Tumor microenvironment
AFP: Alpha-fetoprotein
AFP-L3: Alpha-fetoprotein lens culinaris agglutin-3
DCP: Des-γ-carboxy prothrombin
ICIs: Immune checkpoint inhibitors
